# Intracranial Hemorrhage Due to Secondary Hypertension from Intracranial Large Vessel Occlusion

**DOI:** 10.5811/cpcem.2020.1.44908

**Published:** 2020-03-02

**Authors:** Amit Rawal, Alex Waldman, Omar Saeed, Asif A. Khan

**Affiliations:** * North Florida Regional Medical Center, Department of Emergency Medicine, Gainesville, Florida; † University of Tennessee Health Science Center, Department of Neurology, Memphis, Tennessee; ‡ Zeenat Qureshi Stroke Institute, St. Cloud, Minnesota; § North Florida Regional Medical Center, Department of Vascular and Interventional Neurology, Gainesville, Florida

## Abstract

Simultaneous hemorrhagic and ischemic strokes have been previously reported in the literature. Typically, these occur in patients secondary to dialysis, cerebral amyloid angiopathy, or thrombotic thrombocytopenic purpura.^1^,^2^,^3^ However, this is the unique case of a 62-year-old Asian female who presented with a hemorrhagic stroke suspected to be secondary to refractory hypertension from intracranial large vessel atherosclerotic flow limiting stenosis, with rapid subsequent large vessel occlusion and ischemic stroke. Questions arise such as ideal blood pressure parameters for dual management, timeliness of computed tomography angiography imaging in the emergency department for detection of large vessel occlusion during intracranial hemorrhage, and subsequent selection of treatment plan in the dual-lesion patient population.

## INTRODUCTION

The occurrence of ischemic stroke in those with flow-limiting intracranial atherosclerotic stenosis is well documented and continues to be one of the most common causes of stroke worldwide.^4^,^5^,^6^ The incidence of intracranial atherosclerotic disease is known to be higher in the Asian and African-American populations.^7^ While cases of dual strokes with both hemorrhagic and ischemic components due to other etiology have been previously described, there is a paucity of literature in regard to intracranial hemorrhage (ICH) due to refractory secondary hypertension resulting from intracranial flow-limiting atherosclerotic occlusion. This is a case of a patient who arrived to the emergency department (ED) with a hypertensive-appearing brainstem intraparenchymal hemorrhage who was subsequently found to have severe flow-limiting intracranial stenosis that developed into ischemic cerebral infarction.

## CASE REPORT

A 62-year-old, right-handed, Asian female with history of known refractory hypertension and diabetes mellitus was brought into the ED via emergency medical services (EMS) as a stroke alert soon after experiencing sudden onset of neurologic symptoms. The patient was driving with her husband from San Francisco to Miami when she experienced confusion, slurred speech, and right-sided weakness, which subsequently progressed to unresponsiveness. En route to the ED, EMS reported a blood pressure of 240/120 millimeters of mercury (mmHg) and a glucose level of 133 milligrams per deciliter (mg/dL).

Upon arrival, her Glasgow Coma Scale was seven. Her neurologic examination demonstrated right-sided hemiplegia, left-sided withdrawal to pain, a rightward gaze preference, and reactive pupils. The patient was immediately intubated in the ED for airway protection. Initial vital signs following intubation were as follows: blood pressure 270/159 mmHg; pulse 78 beats per minute; temperature 36.7 degrees Celsius; respiratory rate 18 breaths per minute (on ventilator); pulse oximetry 100% (on 100% fraction of inspired oxygen); and body mass index of 23.5 kilograms per meter squared (kg/m^2^) (reference range 18.5–24.9 kg/m^2^). The patient was started on a nicardipine infusion with a goal systolic blood pressure less than 190–200 mmHg and a propofol infusion for sedation before being taken for imaging.

**Image 1:**
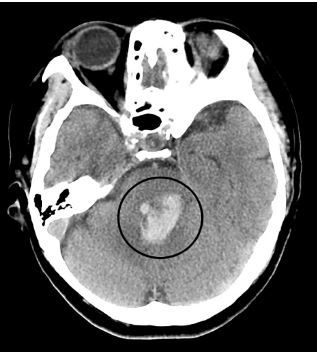
Non-contrast computed tomography brain demonstrating pontine hemorrhage with fourth ventricle involvment (circle).

Initial non-contrast computed tomography (CT) of the brain revealed an ICH that arose in the pons and extended into the fourth ventricle. Her only risk factor for developing the hemorrhagic stroke was the known refractory hypertension. The size of the hemorrhage measured 3.4 centimeters (cm) by 2.0 cm with a volume less than 30 milliliters (mL) (Image 1). An ICH score of three was given, suggesting a 72% mortality. The initial complete blood count and coagulation studies were within normal limits. Intracranial pressure-lowering management was initiated, which included the following: 3% hypertonic saline bolus; 30-degree head of bed elevation; and hyperventilation to target partial pressure of carbon dioxide of 35 mmHg (reference range 35–45 mmHg). Strict systolic blood pressure control to less than 140 mmHg was started with nicardipine infusion as part of the management for ICH. After initiating these measures, an emergent CT angiogram (CTA) was obtained to rule out aneurysm or arteriovenous malformation.

**Image 2:**
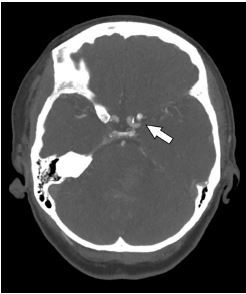
Computed tomography angiogram demonstrating left middle cerebral artery occlusion (arrow).

**Image 3:**
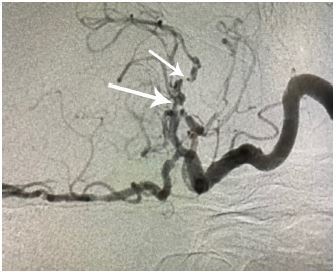
Cerebral angiogram demonstrating near occlusion of left M1 (long arrow) and stenosis of left M2 middle cerebral artery (short arrow).

CTA showed a near occlusion of the left M1 (horizontal proximal) segment of the middle cerebral artery (MCA), which appeared to be chronic (Image 2). The patient was admitted to the intensive care unit with cerebral angiogram scheduled for the following morning to evaluate for left MCA stenosis or cryptogenic malformation. The cerebral angiogram revealed a nearly occluded left M1 MCA with surrounding stenosis involving the proximal M1, multiple M2 branch points, A2 (vertical, post communicating, infracallosal), and A3 (precallosal) anterior cerebral artery branch points consistent with severe intracranial atherosclerosis (Image 3). There was evidence of neovascularization suggesting chronicity of stenosis. Revascularization of the stenotic lesions at this time was considered extraordinarily high risk with the concurrent ICH due to the need for aspirin and clopidogrel during these procedures.

**CPC-EM Capsule**

What do we already know about this clinical entity?

There are established guidelines for blood pressure management, imaging modalities, and definitive treatment options to manage either ischemic or hemorrhagic stroke.

What makes this presentation of disease reportable?

This report discusses the novel presentation of a patient with simultaneous ischemic and hemorrhagic strokes secondary to hypertension from intracranial atherosclerotic stenosis.

What is the major learning point?

Clarity is needed for clinical parameters and treatment options in the dual-lesion patient.

How might this improve emergency medicine practice?

Care will be improved with better-defined management standards and other special consideration, given the unique pathophysiology in dual ischemic and hemorrhagic lesions.

**Image 4:**
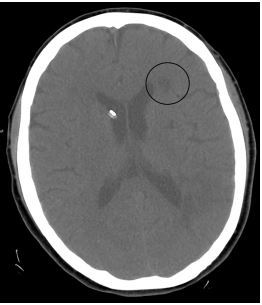
Non-contrast computed tomography brain demonstrating a low attenuated area of the left frontal lobe in a middle cerebral artery watershed area (circle).

On hospital day three, due to concern for developing hydrocephalus, an external ventricular drain was placed, which measured normal intracranial pressure. On hospital day five, increased lethargy was noted while the patient was off of sedative medication. A repeat non-contrast CT of the brain at this time demonstrated a new, low attenuated area involving the high posterior aspect of the left frontal lobe in a MCA watershed area (Image 4). The patient’s neurologic condition continued to decline, and her course was further complicated by aspiration pneumonia. The family ultimately elected to withdraw care, and she passed peacefully on hospital day nine.

## DISCUSSION

Some of the common risk factors for developing intracranial atherosclerotic disease are hypertension, smoking, diabetes mellitus, and hyperlipidemia.^6^ Race and gender also play a major role in the development of extracranial vs intracranial atherosclerotic disease.^8^ Higher incidence of intracranial atherosclerotic disease is seen in Asians from China, Japan, and Korea.^9^ In the Caucasian population, there was a decline in stroke incidence between 1990 and 2005. However, no such decline was seen in the African-American population, whose stroke rates are higher compared to both Caucasian and Southeast Asian groups.^10^

Intracranial arteries are made up of endothelium with smooth muscle cells and an extracellular matrix comprised mostly of collagen and elastin. Stroke due to intracranial atherosclerotic disease is caused by hemodynamic failure, in-situ thrombus from plaque disruption, and/or distal thromboembolism.^11^ Secondary hypertension due to intracranial atherosclerotic disease is a common compensatory mechanism to maintain adequate cerebral perfusion.

Previous reports have discussed the occurrence of simultaneous hemorrhagic and ischemic events in patients secondary to dialysis, cerebral amyloid angiopathy, or thrombotic thrombocytopenic purpura.^1^,^2^,^3^ Examples of events include expanding hemorrhagic intracranial hematoma with subsequent edema that causes compression of adjacent vessels leading to ischemic stroke. There are many other mechanisms for developing ischemic stroke, which can be attributed to embolism, thrombosis, or systemic hypoperfusion.^12^ In this particular patient, however, the ischemic event occurred in the contralateral side of the hemorrhage and in a watershed pattern consistent with hypoperfusion from the underlying stenotic lesion after the lowering of blood pressure to manage the ICH. The concurrent presence of cerebral edema refractory to conventional intravenous treatments may have also contributed to the hemodynamic changes resulting in ischemic stroke.

This patient had severe refractory secondary hypertension due to flow-limiting intracranial atherosclerotic disease. We hypothesize this to be the major contributing factor to her hypertensive ICH. This has implications regarding ideal blood pressure management in the emergent setting. Questions arise whether to decrease blood pressure to limit hemorrhage extension and rebleeding or whether to maintain blood pressure to allow perfusion of the oligemic watershed parenchyma. Ideal blood pressure parameters in patients with both intracranial atherosclerotic disease and intracranial hemorrhage is currently unknown. Current American Stroke Association guidelines for ICH recommend lowering systolic blood pressure to 140 mmHg.^13^ However, patients who have ICH and flow limiting stenosis may benefit from higher blood pressure parameters to maintain adequate cerebral perfusion.

Another important point concerns the timing of CTA in the ED for patients with ICH. The common benefits of routine CTA imaging would include evaluation for aneurysm, malformations, and other intracranial vascular anomalies often seen in spontaneous hemorrhage. CTA also assists in the selection of aneurysmal treatment planning. An additional benefit of CTA in the ED during ICH would be identifying intracranial stenosis, similar to this case, which may be the underlying cause of secondary hypertension.

## CONCLUSION

This is a case of intracranial hemorrhage secondary to refractory hypertension due to severe intracranial atherosclerotic stenosis. Further exploration is needed of the critical questions that arise, such as ideal blood pressure parameters for management, timeliness and routine use of CTA in the ED, and subsequent selection of treatment plan in the dual-lesion patient populations.
